# Exhaled *Mycobacterium tuberculosis* output and detection of subclinical disease by face-mask sampling: prospective observational studies

**DOI:** 10.1016/S1473-3099(19)30707-8

**Published:** 2020-05

**Authors:** Caroline M Williams, Mohamad Abdulwhhab, Surinder S Birring, Elsabe De Kock, Natalie J Garton, Eleanor Townsend, Manish Pareek, Alaa Al-Taie, Jingzhe Pan, Rakesh Ganatra, Anton C Stoltz, Pranabashis Haldar, Michael R Barer

**Affiliations:** a Department of Respiratory Sciences, University of Leicester, Leicester UK; b Department of Engineering, University of Leicester, Leicester UK; c Division of Asthma, Allergy, and Lung Biology, Kings College London, London, UK; d Retrasol Training Solutions, Pretoria, South Africa; e University Hospitals of Leicester NHS Trust, Leicester, UK; f Division of Infectious Diseases, University of Pretoria, Pretoria, South Africa

## Abstract

**Background:**

Tuberculosis remains a global health challenge, with early diagnosis key to its reduction. Face-mask sampling detects exhaled *Mycobacterium tuberculosis*. We aimed to investigate bacillary output from patients with pulmonary tuberculosis and to assess the potential of face-mask sampling as a diagnostic method in active case-finding.

**Methods:**

We did a 24-h longitudinal study in patients from three hospitals in Pretoria, South Africa, with microbiologically confirmed pulmonary tuberculosis. Patients underwent 1 h of face-mask sampling eight times over a 24-h period, with contemporaneous sputum sampling. *M tuberculosis* was detected by quantitative PCR. We also did an active case-finding pilot study in inhabitants of an informal settlement near Pretoria. We enrolled individuals with symptoms of tuberculosis on the WHO screening questionnaire. Participants provided sputum and face-mask samples that were tested with the molecular assay Xpert MTB/RIF Ultra. Sputum-negative and face-mask-positive individuals were followed up prospectively for 20 weeks by bronchoscopy, PET-CT, and further sputum analysis to validate the diagnosis.

**Findings:**

Between Sept 22, 2015, and Dec 3, 2015, 78 patients with pulmonary tuberculosis were screened for the longitudinal study, of whom 24 completed the study (20 had HIV co-infection). *M tuberculosis* was detected in 166 (86%) of 192 face-mask samples and 38 (21%) of 184 assessable sputum samples obtained over a 24-h period. Exhaled *M tuberculosis* output showed no diurnal pattern and did not associate with cough frequency, sputum bacillary content, or chest radiographic disease severity. On May 16, 2018, 45 individuals were screened for the prospective active case-finding pilot study, of whom 20 had tuberculosis symptoms and were willing to take part. Eight participants were diagnosed prospectively with pulmonary tuberculosis, of whom six were exclusively face-mask positive at screening. Four of these participants (three of whom were HIV-positive) had normal findings on chest radiography but had treatment-responsive early tuberculosis-compatible lesions on PET-CT scans, with Xpert-positive sputum samples after 6 weeks.

**Interpretation:**

Face-mask sampling offers a highly efficient and non-invasive method for detecting exhaled *M tuberculosis*, informing the presence of active infection both with greater consistency and at an earlier disease stage than with sputum samples. The approach shows potential for diagnosis and screening, particularly in difficult-to-reach communities.

**Funding:**

Wellcome Trust, CARA (Council for At-Risk Academics), University of Leicester, the UK Medical Research Council, and the National Institute for Health Research.

**Video Abstract:**

Exhaled Mycobacterium tuberculosis output and detection of subclinical disease by face-mask sampling
Dr Caroline Williams introduces the paper on exhaled Mycobacterium tuberculosis output and detection of subclinical disease by face-mask sampling
**YouTube link:**https://youtu.be/G-8majbi2Y0

## Introduction

Improved diagnostics, access to screening, and effective treatment are pivotal in current efforts to control tuberculosis. However, WHO estimate that in 2018, 4·1 million people with tuberculosis were missed from their recorded figures, with mortality known to be high in cases in whom the diagnosis is not made or delayed.^1^ Mathematical models of tuberculosis transmission show that the effect of active case-finding depends on detection of disease in the early or subclinical phase of infection.^2^ Sputum is the principle sample used for microbiological diagnosis;^3^ however, clinical and epidemiological data, including the so-called missing millions,^4^ highlight the limitations of sputum assay both as a diagnostic specimen^1^, ^3^ and as a means of assessing case infectivity.^5^, ^6^, ^7^

With the exception of the guinea pig transmission model devised by Riley, Wells, and colleagues in the 1950s,^8^ *M tuberculosis* output by individuals, in either sputum or aerosol, has been measured in single samples, generally taken at one timepoint or sometimes daily. In the Riley and Wells transmission model, room air from patients with pulmonary tuberculosis was piped to animals housed remotely, and bacterial output could not be linked to specific times or output events.^8^ Variations in *M tuberculosis* output by individuals have been noted in research and in clinical practice,^8^, ^9^, ^10^, ^11^ but natural patterns of exhaled output over time have yet to be ascertained.

**Research in context**

**Evidence before this study**

We searched PubMed with the terms (“patient generated aerosols” OR “bioaerosols” OR “bio-aerosols” OR “face mask sampling”) AND “tuberculosis” AND “human” for articles published in English up to Sept 1, 2019. Our search retrieved 21 articles. As an obligate pathogen, *Mycobacterium tuberculosis* is transmitted by infectious aerosols generated by infected individuals. The quantity of bacteria aerosolised by individuals in one sample is highly variable, as is the infectivity of individuals. The quantity of bacteria aerosolised might not correlate with sputum bacillary burden. Infectious aerosols have been captured from individuals indirectly using resource-intense sampling methods such as the cough-aerosol sampling system, the respiratory aerosol sampling chamber, and large guinea pig transmission platforms. The cough-aerosol sampling system has proven a better predictor of transmission than sputum and shows that the role of larger aerosols and droplets in transmission is unknown. Face-mask sampling has been shown to detect *M tuberculosis* directly from individuals without the need for resource-demanding sampling equipment. However, our search did not identify any studies looking at directly captured *M tuberculosis* bacillary output from individuals over time, nor any attempt to investigate the patterns of variability highlighted in published work. We also did not find any use of aerosol sampling in routine clinical practice as a diagnostic or screening method.

**Added value of this study**

Using modified face masks we have, for the first time, shown *M tuberculosis* output over a full day from 24 patients newly diagnosed with pulmonary tuberculosis. Most patients exhaled consistent *M tuberculosis* levels, but variable high, low, and negative patterns were also noted. No diurnal pattern was apparent. *M tuberculosis* output was not predicted by sputum bacillary content or cough. The ease and frequent positivity of sampling led us to use the mask approach in an active case-finding study. Mask samples detected infection in individuals who would have been missed by sputum analysis (six face-mask-positive sputum-negative *vs* two sputum-positive, among 20 screened) and in individuals with normal findings on chest radiography but early disease identified by PET-CT.

**Implications of all the available evidence**

Understanding aerosol production and its variation is key to understanding and halting tuberculosis transmission. Bacillary burden in aerosol seems to be related poorly to that in sputum, and aerosol samples might prove more useful for recognition of pulmonary infection and transmission risk than traditional markers such as sputum. Face-mask sampling is convenient and readily compatible with routine clinical practice; it shows potential for use in the diagnosis and control of tuberculosis, particularly in settings where access to health care is limited.

Face-mask sampling detects *M tuberculosis* exhaled by patients with pulmonary disease in one sample.^12^ Here, we describe two discrete but thematically related observational studies. In the first study, our primary objective was to ascertain longitudinal variability of *M tuberculosis* output within and between patients and compare these findings with contemporaneous sputum samples taken over 24 h. Associations of *M tuberculosis* output on face masks with corresponding cough frequency and radiological burden of disease were also investigated. In the second study, we did a pilot active case-finding project to establish how face-mask-based testing in the community compared with sputum sampling at one timepoint for early detection of *M tuberculosis* in symptomatic participants.

## Methods

### Study populations

Our first study was a 24-h longitudinal study of patients admitted to one of three hospitals in Pretoria, South Africa (Kalafong Provincial Tertiary Hospital, Tshwane District Hospital, and Steve Biko Academic Hospital) with pulmonary tuberculosis confirmed by either sputum acid-fast bacilli smear or molecular assay (Xpert MTB/RIF; Cepheid, Sunnyvale, CA, USA), clinical symptoms, and radiological abnormalities. Our second study was a 1-day, active case-finding pilot study of inhabitants of an informal settlement in Pretoria, South Africa, in whom one or more symptoms of tuberculosis were reported using the WHO tuberculosis symptom screening questionnaire.^13^ Inclusion criteria for both studies were age 18 years or older, no requirement for oxygen therapy, and untreated (or within 24 h of starting chemotherapy for the longitudinal study).

Participants provided written informed consent in their preferred language. Ethics approval was provided by the Faculty of Health Sciences research ethics committee, University of Pretoria, and the Gauteng Health Department (RET_215_UP03, RET_2017_UP01).

### Procedures

For the longitudinal study, a face mask containing a gelatine membrane sampling matrix was used (appendix p 9). We established the quantitative features of this sampling system in vitro. Direct contamination of face-mask filters with *M tuberculosis* dilutions showed a limit of detection of about 530 copies of IS6110 or 33 colony-forming units. Exposure to nebulised aerosols of *Mycobacterium bovis* BCG for 15 min confirmed the capacity of the gelatine matrix to detect a suitable dynamic range (appendix p 10).

Under direct observation, participants in the longitudinal study wore a face mask containing the gelatine sampling matrix for eight 1-h periods every 3 h over a 24-h period (including during sleep); accumulated sputum was obtained at each interval. Participants also wore an MP3 recorder, configured as previously described for the Leicester Cough Monitor,^14^ to assess cough activity through the sampling period, which was a validated semi-automated analysis. Nocturnal and daytime cough were defined, respectively, for the periods 2300 h to 0500 h and 0500 h to 2300 h. We gathered clinical and demographic data for participants, including routine microbiological investigations (ie, sputum acid-fast bacilli smear microscopy, molecular assay [Xpert MTB/RIF], and liquid culture [BACTEC MGIT 960; Becton Dickinson, Wokingham, UK]). Changes on chest radiography were graded using methods described by Ralph and colleagues.^15^ Duration of symptoms was ascertained by questionnaire.

Exposed gelatine from the face mask was dissolved in sodium hydroxide (1·5 mL of 2% w/v), neutralised with 190 μL 4 mol/L hydrochloric acid, centrifuged at 13 400 × g for 10 min, then the pellet was resuspended in TE buffer (comprising the pH buffer Tris and the cation chelator EDTA [ethylenediaminetetraactic acid]); similar cell suspensions were prepared from sputum after decontamination.^16^ Both suspensions were stored at −80°C. Cells were disrupted by bead-beating and DNA extraction based on the methods of Reddy and colleagues.^17^ *M tuberculosis* sample burden was assayed by IS6110-directed PCR, and a subset was assayed by RD9-directed PCR.^18^, ^19^ Since IS6110 copy number varies between *M tuberculosis* strains, the RD9 (single copy) assay was used to enable comparison of bacterial burden between individuals.

As a background control in the longitudinal study, to assess environmental contamination, we sampled the internal surface of just over a third of the face masks, holders, and transport bags with a moistened swab before use. Eight patients admitted to the same ward as participants with diagnoses other than tuberculosis were also sampled. Molecular water and blank filter controls were used to provide processing and quantification controls. All controls were negative by PCR.

For the active case-finding pilot study, under direct observation, every participant wore a face mask containing a polyvinyl alcohol (PVA) sampling matrix (appendix p 9) for 30 min and provided one contemporaneous sputum sample. The technical development of using an in-house 3D-printed PVA sampling matrix in place of gelatine simplified mask handling and processing is explained further in the appendix (pp 1–9). The exposed PVA matrix was dissolved in 5 mL molecular-grade distilled water (for 5–10 min) and 2 mL of this solution was analysed directly without further processing using the Xpert MTB/RIF Ultra platform. Sputum samples were analysed using Xpert MTB/RIF Ultra, according to the manufacturer's instructions,^20^ at the National Health Laboratory Service in Pretoria, South Africa, which provides clinical microbiology support for the region.

Patients who were face-mask-positive for *M tuberculosis* were assessed by an infectious disease clinician and underwent further diagnostic investigations 6 weeks after the screening event, if sputum analysis did not detect *M tuberculosis* at baseline. These investigations included repeated face-mask and sputum Xpert MTB/RIF Ultra analysis, chest radiography, bronchoalveolar lavage, and PET-CT. This cohort was then reassessed at 20 weeks, 3 months into tuberculosis treatment for some participants. Face-mask samples taken at the 20-week follow-up visit were tested with the DNA-binding dye PMA (propidium monoazide) before Xpert MTB/RIF Ultra analysis to confine positive signals to those derived from DNA within intact and potentially viable cells.^21^

In the active case-finding pilot study, five community patients without symptoms of tuberculosis were used as negative controls. Their masks were all negative by Xpert MTB/RIF Ultra analysis.

Further details of patient sampling and sample processing are provided in the appendix (pp 1–9).

### Statistical analysis

In the absence of previous data using face masks in the ways we describe, both our studies were done using a realistic sample size. For the 24-h longitudinal study, we aimed to recruit 25 individuals to obtain a possible mask sample of 400. For the active case-finding pilot study, we judged a sample size of 20 participants would allow for preliminary comparison of effectiveness between face-mask and sputum samples for detection of *M tuberculosis*. Any differences were intended to inform statistical power calculations for larger studies.

Data were analysed using GraphPad Prism (version 7), Excel (Microsoft 2010), and SPSS (version 22). The Shapiro-Wilk test showed that cough frequency and IS6110 copies in face-mask and sputum samples were non-normally distributed. Therefore, median (IQR) values are presented, and comparisons were made using the Mann-Whitney *U* test or Wilcoxon signed rank test. We used Spearman's ranking to test for correlations. Cohen's kappa coefficient test was used to assess agreement between face-mask and sputum results.

### Role of the funding source

The funders had no role in study design, data collection, data analysis, data interpretation, or writing of the report. The corresponding author had full access to all data in the study and had final responsibility for the decision to submit for publication.

## Results

**Figure 1:**
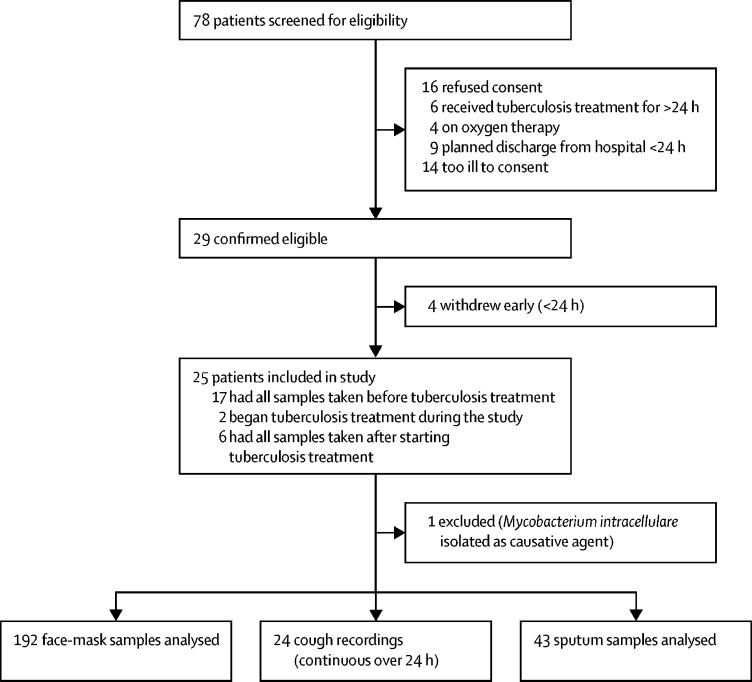
Longitudinal study profile Patients all had pulmonary tuberculosis confirmed by sputum acid-fast bacilli smear or molecular assay with Xpert MTB/RIF.

Between Sept 22, 2015, and Dec 3, 2015, 78 patients with pulmonary tuberculosis were screened for the 24-h longitudinal study, of whom 33 were ineligible and 20 declined to participate either before or after study enrolment (figure 1). One smear-positive participant from whom *Mycobacterium intracellulare* was cultured was excluded. Thus, 24 participants completed the study; characteristics of this cohort are shown in table 1. For 16 patients, all samples were taken before they began tuberculosis treatment, with the remaining eight patients receiving one dose of chemotherapy either before or during their respective sampling periods. All participants in the longitudinal study were of black African ethnic origin and were admitted to hospital with microbiologically confirmed pulmonary tuberculosis. The median age of the cohort was 35 years (IQR 26–42) and 14 (58%) participants were female. HIV co-infection was identified in 20 (83%) participants, with a median CD4 count of 35 cells per μL (IQR 17–74); four (20%) patients with HIV co-infection were receiving antiretroviral therapy at enrolment.

**Table 1:** Characteristics of patients with pulmonary tuberculosis in the longitudinal study, by pattern of face-mask output

		Consistent producers*(n=16)	**High variable producers (n=2)**	**Low variable producers**†**(n=4)**	**Negative producers (n=2)**‡	**Whole cohort (n=24)**§
Age, years		34·0 (28·8–40·5)	30·0 (25·0–35·0)	34·0 (24·3–49·0)	51·0 (45·5–57·5)	35·0 (25·8–42·3)
Male		6 (37%)	2 (100)	1 (25%)	1 (50%)	10 (42%)
Female		10 (63%)	0 (0%)	3 (75%)	1 (50%)	14 (58%)
HIV-positive		15 (94%)	2 (100%)	3 (75%)	1 (50%)	20 (83%)
CD4 count, cells per μL		26 (16–46)	167 (132-201)	44 (31-105)	66	35 (17-74)
Symptom duration before enrolment, weeks		4·0 (3·0–8·5)	4·0 (3·0–5·0)	4·0 (3·8–4·0)	3·0 (3·0–3·0)	4·0 (3·0–6·5)
Chest radiography severity grade¶		65·0 (30·0–78·0)	58·0 (49·0–66·0)	68·0 (51·0–79·0)	45·0 (42·5–47·5)	60·0 (35·0–70·0)
Cavitation		6 (40%)	1 (50%)	2 (50%)	1 (50%)	10 (46%)
Duration of tuberculosis treatment						
	No treatment	11 (69%)	2 (100%)	2 (50%)	1 (50%)	16 (67%)
	24 h	3 (19%)	0 (0%)	2 (50%)	1 (50%)	6 (25%)
	<24 h	2 (12%)	0 (0%)	0 (0%)	0 (0%)	2 (8%)
Produced sputum during the study		13 (81%)	2 (100%)	2 (50%)	2 (100%)	19 (79%)
Volume of sputum, mL		4·3 (2·0–6·0)	95·0 (50·0–140·0)	6·8 (6·4–7·1)	9·5 (5·0–14·0)	5·0 (2·1–6·4)
Cough frequency, coughs per 24 h		406 (241–507)	1383 (825–1942)	337 (165–893)	322 (193–452)	466 (234–551)
Patient's perception of cough severity‖		6·9 (4·9–9·5)	8·4 (7·6–9·2)	7·3 (3·4–10·0)	2·0 (NA)	6·9 (4·5–9·8)
Sputum Xpert MTB/RIF grading						
	Negative	1 (6%)	0 (0%)	0 (0%)	0 (0%)	1 (4%)
	Very low	1 (6%)	0 (0%)	0 (0%)	0 (0%)	1 (4%)
	Low	6 (38%)	0 (0%)	1 (25%)	1 (50%)	8 (33%)
	Medium	3 (19%)	1 (50%)	2 (50%)	0 (0%)	8 (33%)
	High	5 (31%)	1 (50%)	1 (25%)	1 (50%)	6 (25%)
Sputum acid-fast bacilli grading						
	Negative	6 (46%)	1 (50%)	2 (67%)	NA	9 (50%)
	Scanty	1 (8%)	0 (0%)	0 (0%)	NA	1 (8%)
	1+	3 (23%)	0 (0%)	0 (0%)	NA	3 (12%)
	2+	1 (8%)	0 (0%)	0 (0%)	NA	1 (8%)
	3+	2 (15)	1 (50)	1 (33)	NA	4 (22%)
Time to positivity on liquid culture, days		12 (11–13)	12 (8–16)	8 (6–10)	NA	12 (8–13)

Data are median (IQR) or n (%). NA=not available.

* Missing data for sputum acid-fast bacilli grade (n=3), liquid culture (n=3), perception of cough (n=1), chest radiography severity grade (n=1), and cavitation (n=1).

† Missing data for sputum acid-fast bacilli grade (n=1) and liquid culture (n=2).

‡ Missing data for sputum acid-fast bacilli grade (n=2), liquid culture (n=2), and perception of cough (n=1).

§ Missing data for sputum acid-fast bacilli grade (n=6), liquid culture (n=7), perception of cough (n=2), and chest radiography severity grade (n=1), and cavitation (n=1).

¶ Chest radiography severity grade based on extent of disease and presence of cavitation (range 0–140).^14^

‖ Measured using visual analogue score (range 1–10).

**Figure 2:**
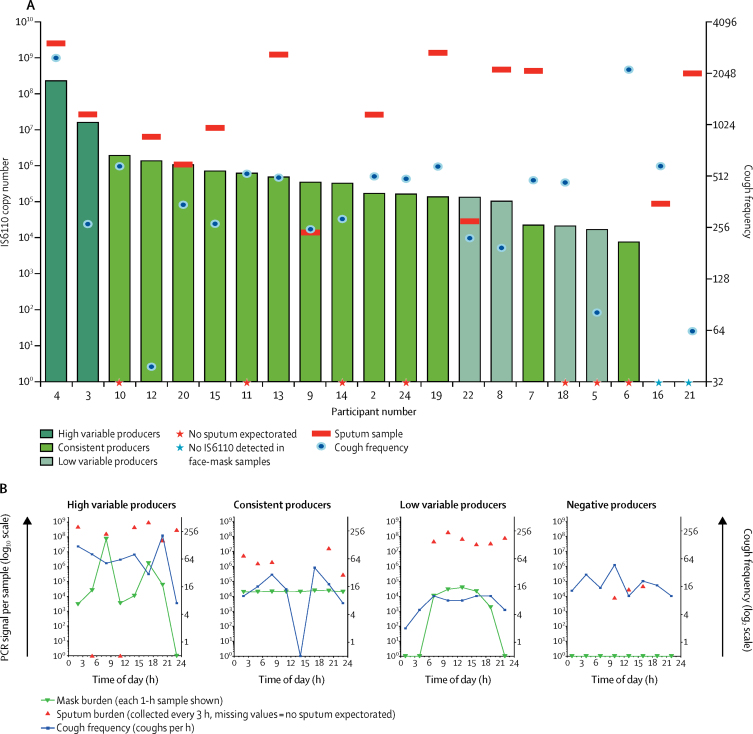
Mycobacterial output in face-mask and sputum samples, with cough counts over 24 h Cumulative *Mycobacterium tuberculosis* output in 21 patients (A); three of 24 patients who completed the study were excluded from this analysis because of an error in the processing of their sputum samples. Pattern of *M tuberculosis* output and cough count over 24 h (B).

A 1-h face-mask sample was readily obtained from 24 participants during each of the 3-h sampling periods, for a total of 192 face-mask samples. *M tuberculosis* was detected by PCR in one or more dissolved gelatine filters from 22 participants, with 17 individuals positive for *M tuberculosis* in every face-mask sample (appendix p 11). Based on the variability and amount of *M tuberculosis* detected, three patterns of *M tuberculosis* longitudinal output were recognised (figure 2A). A consistent pattern described 16 patients in whom hourly outputs varied predominantly within a ten-fold range (median 3·4 × 10^5^ IS6110 copies per 24 h; appendix p 12). By contrast, six patients had variability in their longitudinal output over at least two orders of magnitude. Within this group, a distinction between high variable output (median 1·2 × 10^8^ IS6110 copies per 24 h; n=2) and low variable output (median 6·0 × 10^4^ IS6110 copies per 24 h; n=4) was made (appendix p 11). These patterns were not associated with any clear diurnal cycle (figure 2B; appendix p 11).

No association was seen between cough frequency and face mask *M tuberculosis* output, either for cumulative 24 h measurements or at an hourly level (figure 2A; appendix p 13). Cough frequency declined significantly during sleep but was not associated with any corresponding change in face mask *M tuberculosis* output; indeed, continued *M tuberculosis* output was shown in 11 samples from sleeping individuals in the absence of any recorded coughs (appendix p 13).

We found no association between total face-mask-detected *M tuberculosis* output and radiological measures of disease extent, including presence of cavities on chest radiography (p=0·52). Further, no association was noted between face-mask-detected *M tuberculosis* output and measures of bacterial burden (acid-fast bacilli, Xpert MTB/RIF grade, or time to positivity on liquid culture) in baseline sputum samples (table 2). These findings remained consistent after the repeating analysis using RD9-normalised *M tuberculosis* signals from face-mask and sputum samples.

**Table 2:** Potential predictors of bacterial burden in longitudinal sputum and face-mask samples over 24 h

	**24-h face-mask IS6110 output**		**24-h face-mask RD9 output**	
	Correlation coefficient (95% CI)	p value	Correlation coefficient (95% CI)	p value
Age	−0·26 (−0·61 to 0·17)	0·21	−0·13 (−0·52 to 0·30)	0·53
Gender	..	0·89	..	0·47
HIV status	..	0·48	..	0·68
CD4 count*	−0·28 (−0·65 to 0·20)	0·23	−0·11 (−0·53 to 0·36)	0·65
Duration of symptoms, weeks	0·18 (−0·26 to 0·55)	0·41	0·11 (−0·31 to 0·48)	0·56
Chest radiography grade†	−0·22 (−0·59 to 0·23)	0·32	−0·07 (−0·48 to 0·36)	0·75
Presence of cavitation on chest radiography†	..	0·52	..	0·38
Sputum acid-fast bacilli grade‡	−0·08 (−0·54 to 0·41)	0·75	−0·02 (−0·49 to 0·47)	0·95
Sputum Xpert MTB/RIF grade	0·02 (−0·40 to 0·43)	0·92	−0·04 (−0·45 to 0·37)	0·84
Sputum culture§	0·14 (−0·38 to 0·59)	0·58	0·07 (−0·44 to 0·54)	0·79
Cough frequency	0·10 (−0·33 to 0·50)	0·63	0·07 (−0·35 to 0·47)	0·73
Patient's perception of cough severity, visual analogue scale¶	0·28 (−0·18 to 0·63)	0·21	0·31 (−0·15 to 0·65)	0·53
Started tuberculosis treatment	..	0·68	..	0·91
24-h sputum PCR signal content	0·17 (−0·30 to 0·57)	0·47	0·15 (−0·31 to 0·56)	0·49

Data represent the ability of variables to predict levels of IS6110 and RD9 signals detected in face-mask samples over 24 h, analysed by Spearman's correlation for continuous variables and the Mann-Whitney *U* test for categorical data.

* CD4 count was recorded for all 20 HIV-positive patients.

† Chest radiography grade and presence of cavitation was recorded for 23 patients.

‡ Sputum acid-fast bacilli grade available for 17 patients.

§ Sputum culture results available for 16 patients.

¶ Visual analogue scale recorded for 22 patients.

Sputum was produced spontaneously by 18 (75%) of 24 participants at 51 (27%) of 192 possible timepoints, with between one and eight samples produced and total volumes of 1–185 mL (figure 2A). Eight sputum samples from three patients were excluded because of processing errors. Two individuals produced no face-mask-detected *M tuberculosis* but did produce one or more positive sputum samples (figure 2A). No diurnal pattern was discernible in either sputum production or *M tuberculosis* content per sputum sample (figure 2B; appendix p 11). No association was noted between sputum and face-mask *M tuberculosis* output, either over 24 h (p=0·065) or within the patterns of longitudinal output we have described (p=0·36). Overall, total 24-h *M tuberculosis* sputum content was significantly higher than that detected in mask samples (p=0·018), but this finding was not universal because higher total output was detected in face-mask samples from two participants and three individuals produced no sputum. In the 43 sampling periods that provided both a sputum sample and face-mask sample, *M tuberculosis* was detected in at least one sample type in all cases. Discordant results were obtained on 12 occasions, with seven positive for the sputum sample alone and five positive for the face mask alone.

Sputum sensitivity (when a sample was produced) was 88·3% (38 of 43). However, sputum was only available at 43 of 184 assessable sampling timepoints, giving an overall sensitivity of 20·7% (38 of 184) at any given timepoint over 24 h, compared with a sensitivity of 86·5% (166 of 192) for face-mask sampling.

**Figure 3:**
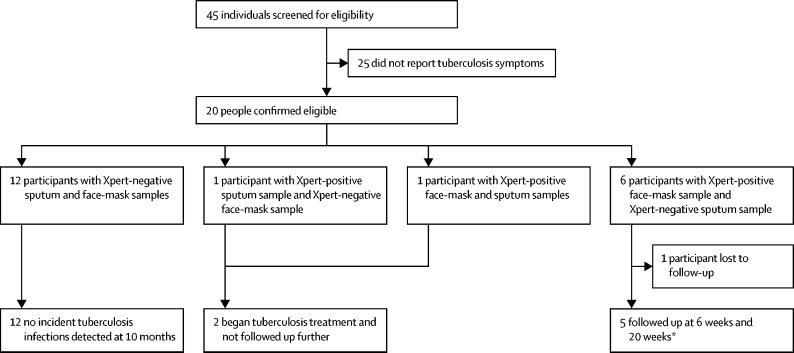
Active case-finding pilot study profile Patients were all living in an informal settlement and were screened with the WHO tuberculosis symptom screening questionnaire. Xpert=molecular assay Xpert MTB/RIF Ultra. *Follow-up at 6 weeks consisted of repeat Xpert analysis of face-mask and sputum samples, bronchoalveolar lavage with Xpert analysis, chest radiography, and PET-CT. Follow-up at 20 weeks consisted of repeat Xpert analysis of face-mask and sputum samples, propidium monoazide and Xpert analysis of face-mask samples, and repeat PET-CT.

On May 16, 2018, 45 individuals were screened for the prospective active case-finding pilot study, of whom 20 had tuberculosis symptoms and were willing to take part in the study (figure 3). The median age of participants was 31·5 years (IQR 19·0–47·0), and eight (40%) were female. 12 patients were sputum-negative and face-mask-negative at baseline. Among these 12 participants, no incident cases of tuberculosis arose at 10 months of follow-up.

**Figure 4:**
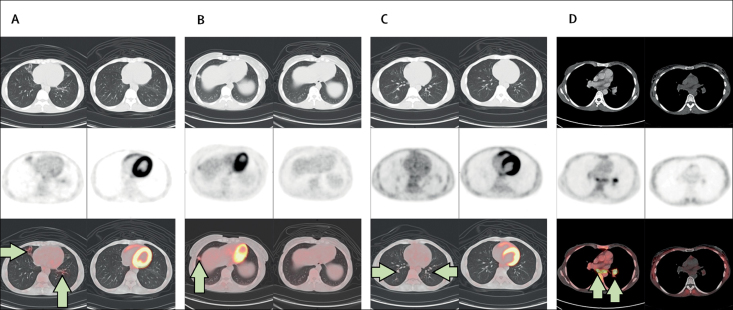
PET-CT images from four patients investigated for tuberculosis Four patients who were face-mask-positive but sputum-negative for *Mycobacterium tuberculosis* at screening were followed up for 20 weeks. Matched 6-week (left) and 20-week (right) images for each patient (A–D). CT images are shown in the upper windows, PET scans in the middle windows, and the fused dataset in the lower windows. (A–C) Arrows show parenchymal lung changes (tree and bud) with associated increased ^18^F-fluorodeoxyglucose uptake on the 6-week scan, which resolved completely at 20 weeks. (D) Arrows show mediastinal and left hilar lymph nodes, which were active on the 6-week scan and resolved completely at 20 weeks.

The remaining eight participants had *M tuberculosis* detected using Xpert MRB/RIF Ultra at the baseline visit, of which six cases were detected by face mask alone, one by sputum only, and one by both methods. The two patients who were sputum-positive were treated for tuberculosis and not followed up further. Of the six participants who were exclusively face-mask-positive, one was lost to prospective follow-up. Among the five participants who were followed up, four became sputum-positive at 6 weeks; the remaining patient had consistently negative conventional investigations for tuberculosis at follow-up. Four of the five patients were HIV-positive, with two receiving antiretroviral therapy. Among the four patients who became sputum-positive at 6 weeks (three of whom were HIV-positive), no evidence on chest radiography was seen for active tuberculosis at 6 weeks; however, early parenchymal changes were noted on PET-CT and were characterised by nodularity and tree-in-bud change, consistent with bronchiolitis (figure 4). Typically, lung parenchymal changes were non-specific and confined to the middle and lower lobes of the lung. All active lung abnormalities resolved after 3 months of anti-tuberculous therapy, which was associated with reversion of Xpert MTB/RIF Ultra in sputum and face-mask samples (table 3).

**Table 3:** Investigations for tuberculosis disease in the active case-finding pilot study

	**Initial screening**		**6-week follow-up**						**20-week follow-up**			
	Sputum sample	Face-mask sample	Sputum sample	Face-mask sample	Chest radiography	Bronchoalveolar lavage result	PET-CT	Outcome	Sputum sample	Face-mask sample*	PET-CT	Outcome
2	−	+	−	+	−	−	−	Treatment withheld and observed	−	−	−	Alternative diagnosis made
3	−	+	+	+	−	+	+	Tuberculosis treatment commenced	−	−	−	Completed treatment
7	−	+	+	+	−	+	+	Tuberculosis treatment commenced	−	−	−	Completed treatment
13	−	+	+	+	−	−	+	Tuberculosis treatment commenced	−	−	−	Completed treatment
17	−	+	+	+	−	−	+	Tuberculosis treatment commenced	−	−	−	Completed treatment

Investigations were done in five participants (patient IDs 2, 3, 7, 13, and 17) who were exclusively face-mask-positive. Sputum and face-mask samples were analysed using the Xpert MTB/RIF Ultra platform.

* Face-mask samples at 20-week follow-up were treated with propidium monoazide before Xpert MTB/RIF Ultra analysis.

The fifth participant with a positive face-mask sample at baseline but with negative findings for tuberculosis at follow-up had completed a course of tuberculosis treatment 6 months before the study. Xpert MTB/RIF Ultra results were negative after PMA testing of a face-mask sample at 20 weeks, which suggests false-positive detection of DNA remnants by the face mask at previous visits.

## Discussion

Using a face-mask-sampling approach, we have provided first insights into daily *M tuberculosis* output exhaled by patients with tuberculosis. Our findings show novel patterns of bacterial output and highlight the discordance between *M tuberculosis* output in breath and sputum. In particular, we have shown that over a 24-h sampling period, applied to patients with established sputum-positive disease, *M tuberculosis* is detected at least four times more frequently with face masks than in sputum samples. Moreover, we have shown the clinical effect of this increased level of detection in an active case-finding pilot study in which face-mask sampling achieved microbiological diagnosis of active tuberculosis from one sample up to 6 weeks before conventional sputum diagnosis, providing scope to interrupt possible transmission at an earlier stage of disease.

To the best of our knowledge, we have presented the first longitudinal data of exhaled *M tuberculosis* over 24 h in patients with pulmonary tuberculosis. Despite clinical and radiological features to suggest infectiousness, a few patients were identified who exhaled no detectable *M tuberculosis* over 24 h. By contrast, a similar proportion of patients in our cohort were identified as high variable producers, generating both the highest peak and total *M tuberculosis* output. These observations support the view that transmission risk between patients with pulmonary tuberculosis is heterogeneous. Although we are unable to prove greater infectiousness of the high variable producer group, it is notable that the proportion of our cohort with this phenotype (two of 24) is comparable with the findings of Escombe and colleagues,^9^ who reported that 8·5% of patients in an HIV cohort were highly infectious to guinea pigs in a Riley design facility.

We did not record any evidence of a diurnal pattern in *M tuberculosis* production. Although hospitalisation could have disrupted natural rhythm, the diurnal pattern of cough that has been reported in previous studies was seen.^14^, ^22^, ^23^

We saw no evidence that any of the traditional clinical markers of infectivity (eg, cough frequency, radiological characteristics, and bacillary content of sputum) were associated with either the pattern or quantity of longitudinal *M tuberculosis* output detected by face-mask sampling. Particular attention was given to discordance between detection of *M tuberculosis* in face-mask and sputum samples. For 43 sampling periods in which both face-mask and sputum samples were available, *M tuberculosis* levels were not correlated in pairwise comparisons and neither were cumulative 24-h *M tuberculosis* outputs. This absence of an association was also seen for *M tuberculosis* quantified by sputum microscopy, Xpert MTB/RIF grade, and time to positivity on liquid culture.

Other studies using particle size selective aerosol capture methods have reported discordance between *M tuberculosis* aerosol burden and both sputum bacillary content and disease grade on chest radiography.^5^, ^24^, ^25^ Although current guidance retains a focus on sputum and chest radiographic analyses, Jones-Lopez and colleagues^25^ showed a significant association between household transmission and actively elicited cough-aerosol *M tuberculosis* output, supporting the need to look beyond sputum and other established indices in the assessment of transmission risk. We emphasise that our findings from this small-scale study cannot be considered to undermine the established view that transmission risk relates to bacillary burden in sputum at the population level. However, both the cough-aerosol sampling system studies (directly)^5^, ^25^ and our data (by implication) do show discordance at the individual level.

We noted no relation between cough frequency and longitudinal face-mask *M tuberculosis* output associated with individual sampling periods or cumulatively over 24 h. This dissociation was emphasised by our observation of 11 periods in six sleeping individuals during which time positive face-mask samples were obtained but no coughs were recorded, and six periods characterised by high cough frequency (more than five times the median cough frequency for the whole cohort) and no exhaled *M tuberculosis* output. It is also notable that, in respect of cough frequency, our cohort seems broadly similar to others studied^26^ (appendix p 14).

Associations between cough and *M tuberculosis* aerosol output have been reported in two study systems. Fennelly and colleagues^27^ reported on aerosols actively elicited by coughing over two 5-min periods onto agar plates in size-selective Andersen samplers, whereas Patterson and colleagues^24^ described a cough chamber with multiple particle size-determining features linked to both culture and molecular readouts. In the first case, coughing was an essential part of the sampling procedure whereas, in the second case, spontaneous *M tuberculosis* output showed modest association with coughs recorded over 1 h. We do not know the particle size distribution contributing to the *M tuberculosis* signals detected by face-mask sampling, and inclusion droplets larger than those collected in the Fennelly and Patterson samplers could account for our result.

In our active case-finding pilot study, we cannot say whether DNA signals measured came from intact or disrupted *M tuberculosis* cells. Signals were quantified from pelleted whole bacteria, which makes signals coming from free DNA less likely, although we cannot comment on morphology or viability of these cells. Furthermore, we note that signals from viable bacteria reported in our previous face-mask sampling study^12^ are comparable to those detected in our active case-finding pilot study. Use of PMA to eliminate signals from residual DNA from dead cells might allow better discrimination in future.

The role of salvia or airway secretions in face-mask outputs is unknown, particularly in samples taken during sleep. Indeed, the importance of airway secretions and salivary sputum have been long considered,^28^ and it has been suggested that aerosolisation of bacilli or their components is more likely from airways with low volume and watery secretions.^10^

Nonetheless, our longitudinal study is the first to objectively and systematically record longitudinal frequency of spontaneous cough linked to *M tuberculosis* output in real time. Moreover, although the roles of larger expectorated droplets and airway secretions are unknown,^10^, ^28^, ^29^ bacillary content in face-mask samples possibly reflects the overall potential for airborne transmission.

In our longitudinal 24-h sampling study, face-mask sampling detected *M tuberculosis* more than four times more frequently than in sputum analyses. The main reason for this increase was that face-mask samples were obtained at every timepoint, whereas sputum was only available for just over a quarter of the sampling periods. In the active case-finding pilot study, although all patients produced sputum, face-mask sampling detected pulmonary tuberculosis with greater sensitivity than did both sputum and chest radiography, with sampling at one timepoint. Importantly, face-mask sampling detected disease at an earlier stage, supporting assessment in larger community-based studies as a means to affect onward transmission of infection and prospective tuberculosis control.^1^ Furthermore early detection (and subsequent treatment) of pulmonary tuberculosis, facilitated by face-mask sampling, could be a large benefit for preventing pulmonary impairment after tuberculosis, a major source of global pulmonary morbidity and disability.^30^

Our detailed longitudinal characterisation of the face-mask-screened cohort provides strong evidence that participants with compatible symptoms of tuberculosis and a positive face-mask sample had active disease. Treatment was withheld in participants who were exclusively face-mask-positive at baseline until corroborating evidence for active tuberculosis was available. Sputum sampling at 6 weeks identified *M tuberculosis* in four of five patients who had negative findings at baseline, suggesting at least some disease progression over this period to achieve this endpoint. Radiological assessment at this stage identified only subtle abnormalities in lung parenchyma on PET-CT, suggesting that 6 weeks after mask positivity, disease was still at a very early stage. Although these observations support that sputum analysis can be positive with radiologically early disease, they emphasise the potential of even earlier intervention with face-mask sampling. Further studies offer the prospect to do detailed characterisation of the earliest stages of active tuberculosis to inform the pathophysiology of incipient disease.

The main limitation of both our studies is the absence of an a-priori power calculation to inform sample size estimates for each part of the study. Previous data in this area were unavailable to support a reliable sample size calculation. We emphasise that our studies are proof-of-concept and intended to provide observational data to inform further work. We accept that although the absence of an association between face-mask and sputum *M tuberculosis* output seen in the first study is concordant with other studies,^10^, ^24^ the possibility of insufficient study power cannot be excluded. It is also notable that since face masks could not be worn continuously, total *M tuberculosis* face-mask output can only be estimated and is liable to error, in view of the variability in output that was seen. By contrast, total sputum bacillary burden reflects all sputum samples produced in the 24-h period. This fact could have contributed to the poor correlation between the two measures and might also have underestimated the total 24-h mask output in some of the participants. In the active case-finding pilot study, our findings are clear and support the need for studies in larger cohorts to validate our findings and investigate the potential use of the face-mask system as both a screening method and for clinical diagnosis of tuberculosis and possibly other lower respiratory tract infections.

Our studies were undertaken in a real-life setting in South Africa and, therefore, our cohorts reflect the disease burden and diagnostic practices of this setting. The HIV co-infection rates in both studies were high, and hospitalised individuals tended to have advanced disease; thus, caution should be exercised in extrapolating our findings to other settings.

Our decision to use the WHO symptom screening algorithm to target screening for tuberculosis in the active case-finding pilot study was consistent with conventional approaches to screening in a real-life setting.^1^ We recognise the relatively poor sensitivity of the WHO questionnaire,^1^ and it is likely that the prevalence of unrecognised active tuberculosis will have been underestimated. Future studies should investigate the frequency of face-mask and sputum positivity among cohorts with few or no tuberculosis-related symptoms. Detection of *M tuberculosis* by DNA assay requires consideration. For the first study, we chose the strain-variable multicopy gene target IS6110 to provide sensitivity. However, although this target does not affect within-patient trends, it does complicate between-patient comparison because copy numbers vary up to 20-fold between *M tuberculosis* strains. Such variation would not substantially affect our conclusions; moreover, normalisation to RD9 did not alter the ranking of our results.

Although we have not linked our detection of exhaled *M tuberculosis* signals to transmission, such an association is plausible (if not at least necessary) and we suggest that face-mask sampling has potential as a method to inform individual transmission risk. Further studies to characterise links between face-mask sample *M tuberculosis* burden and transmission are needed in settings with both high and low incidence of tuberculosis.

Face-mask sampling offers a new approach to understanding and diagnosing tuberculosis. It is simple, clinically compatible, can detect other target organisms,^31^ reliably yields a sample, and seems superior to sputum samples for detection of early tuberculosis disease. The approach shows potential for diagnosis and screening, particularly in difficult-to-reach communities. We are currently engaged in larger scale and community-based studies to determine the potential of this approach to enhance early diagnosis and transmission control in tuberculosis.

**Contributors**

CMW had the idea for the study and contributed to data collection, laboratory processing of samples, data interpretation, statistical analysis, and writing of the report. MA designed laboratory extraction methods. SSB contributed to cough monitor design and cough data analysis, manuscript review, and writing of the report. EDK contributed to project management and manuscript review. NJG contributed to data interpretation, manuscript review, and writing of the report. ET contributed to sample collection in the active case-finding study and laboratory processing of samples. MP contributed to manuscript review and writing of the report. AA-T and JP had the idea for the polyvinyl alcohol sampling matrix and contributed to its development and supply. ACS supervised the project and contributed to manuscript review. PH contributed to data interpretation, statistical analysis, and manuscript review. MRB had the idea of mask sampling and the idea for the study, and contributed to data interpretation, manuscript review, and writing the report. RG contributed to PET-CT analysis and manuscript preparation and review.

**Declaration of interests**

We declare no competing interests.
