# Supplementary material for: Exhaled Mycobacterium tuberculosis output and detection of subclinical disease by face-mask sampling: prospective observational studies
Source: Lancet Infect Dis. 2020 May;20(5):607–17. doi: 10.1016/S1473-3099(19)30707-8 (PMC7191268; doi:10.1016/S1473-3099(19)30707-8)
Supplement: Supplementary appendix [file mmc1.pdf]

THE LANCET Infectious Diseases

## Supplementary webappendix

This webappendix formed part of the original submission and has been peer reviewed. We post it as supplied by the authors.

Supplement to: Williams CM, Abdulwhhab M, Birring SS, et al. Exhaled *Mycobacterium tuberculosis* output and detection of subclinical disease by face-mask sampling: prospective observational studiest. *Lancet Infect Dis* 2020; published online Feb 18. https://doi.org/10.1016/S1473-3099(19)30707-8.

**Supplemental Data**

Detailed Methods - Study one

Mask and Sputum Sampling

Each participant wore a modified FPP1 face mask containing a gelatine filter (pore size 0.3µm, Sartorius, Germany. Figure S1). Subjects were specifically not required to perform any vocal manoeuvres and were allowed to cough, talk, laugh or sleep as desired. If they needed to expectorate then they were asked to lift the mask briefly, after coughing, to expectorate into a sputum collection pot.

Each participant underwent mask sampling for an hour every three hours and were provided with a new sputum pot at every three hour interval (commencing with the hour of mask sampling) and encouraged to collect whatever they expectorated spontaneously for the 24 hour study duration. We fully acknowledge that material collected in the mask is not formally consistent with accepted definitions of the term aerosol and that the signals we detect may include bacilli transferred in larger droplets but that the bacillary content likely reflects the overall potential for airborne transmission.

Subjects were observed to ensure that the mask was worn for the whole hour of sampling and participant behaviour was recorded, including anytime the mask was directly in front of the mouth. Sleep was documented if the participant had their eyes closed for >10 minutes and were not obviously rousable by noise, resting was noted if the participant had their eyes closed for <10 minutes at a time and/ or had their eyes open but not engaging in any activity such as talking, eating, reading etc. Other activities such as eating, washing, reading and talking were documented.

The reasons that participants declined from taking part in this study was diverse. The majority (9) did not want the inconvenience of repeated sampling over the 24 hours, 2 were

too distressed by their new diagnosis of HIV and TB to take part, 2 felt to hot and unwell to undergo sampling, 1 did not think they would be in hospital within the next 24 hours and 2 declined to give a reason. Two of the four that withdrew early did so because they did not want their sleep to be disturbed further, 1 said they felt more unwell and did not want to participate any longer and 1 participant took their own discharge from hospital against medical advice.

Initial processing of Mask and Sputum samples

Gelatine filters were dissolved in 1.5mls of 2% w/v NaOH and incubated at room temperature for 15 minutes before neutralising with 190µl 4M HCL. Samples were agitated by hand at 0 and 8 minutes. The dissolved filter was then centrifuged at 13,400 x*g* for 10 minutes, the supernatant removed and the pellet overlaid with 100µl of TE buffer (20mM Tris and 2mM EDTA pH 8.0), prior to storage at -80°C.

Sputum was decontaminated in accordance with Turapov and colleagues(1), following centrifugation at 4,000 xg for 20 minutes the pellet was re-suspended in 1ml of the supernatant, passed through a 23G blunt needle 10 times and centrifugation at 13,400xg for 10 minutes. The supernatant was removed and the pellet overlaid with 100µl of TE buffer (20mM Tris and 2mM EDTA pH 8.0) prior to storing at -80°C.

Extraction and quantification of DNA from bacterial pellets

Bacterial pellets from the mask and sputum samples underwent the same in-house extraction method modified from that outlined by Reddy and colleagues(2). 100µl Chelex-NP40 (50% w/v Chelex-100, 1% w/v Non-idet P40, 1% w/v Tween 20) was added to the defrosted bacterial pellet along with 0.3g glass beads (150-212µm Sigma-Aldrich USA). The sample was then homogenised in a Fast Prep at 6.5 m/s for 45 seconds and ice incubated for 5

minutes. This homogenisation step was repeated 3 further times prior centrifugation at 13,400xg for 2 minutes. 200µl of the supernatant was heated at 95°C for 30 minutes prior to removal from the CL3.

Copy numbers of IS6110 were quantified in each sample by real-time q-PCR run on a Rotor-Gene (Qiagen UK) using a TaqMan IS6110 assay outlined by Akkerman and colleagues as 'in-house TaqMan -10' (3). Real-time PCR signals were analysed by Rotor-Gene 6000 Series Software 1. The functions "slope correct" and "ignore cycles" were applied to analyses. In accordance with Dorak and colleagues only runs with correlation coefficients ($R^2$ ) and reaction efficiencies above 0.99 and 0.8 respectively were included for analysis(4). Technical replicates were undertaken in triplicate and considered reproducible if the difference in cycle threshold (Ct) was less than 1.

As differences in IS6110 copy number between bacterial strains limits confidence in comparative analysis, a subset of samples for each participant was further analysed using the Mtb-specific RD9 DNA sequence, single copy gene(5). Quantification by real-time q-PCR of this gene was undertaken using the RD9 specific primers designed by Chae and colleagues (5) under the following conditions. The 25µl reaction volume consisted of 12.5µl SensiFAST SYBR No-ROX (Bioline, UK), 1µl (10µM) of RD9 specific forward and reverse primers (5), 1µl of DNA template and 9.5µl of molecular-grade water (Hyclone™, UK filter sterilised 0.1µm). The cycling conditions were: 1 cycle at 52°C for 2 minutes, 1 cycle at 95°C for 15 minutes, 40 cycles at 95°C for 20 seconds, 61.5°C for 30 seconds, 72°C for 20 seconds and 80°C for 20 seconds with acquisition on FAM/Green channel (470nm). The melting conditions were: 72°C to 95°C rising 1°C per cycle. Comparative statistical analyses have been assessed using both IS6110 copy numbers and genome copy number derived using the ratio between IS6110 and RD9 (Table S1).

Cough Sampling

A Leicester Cough Monitor (LCM) was worn by each participant for the 24 hours of the study. As described by Birring and colleagues, the LCM consists of an MP3 recorder (Sony ICD PX333) worn at the participant's waist in protective bag connected to a clip microphone (Philips LFH9165) that is worn as close to the sternoclavicular joint as possible(6). Recordings are analysed using specialised semi-automated software as previously described, that anonymised sounds and quantified coughs both as single events or cough bouts(6). The LCM position was checked every 3 hours throughout the study. As well as being well validated for use in patients with many respiratory conditions including TB (6-9) the LCM was validated for use with mask sampling prior to this study.

A subset of the recordings were validated by a second member of the research team, who was 'blinded' to the patient information and the primary analysis of the recording. There was good agreement in the subset reviewed by both members of the research team with an Intra-Class Correlation of R=0.99 (CI 0.996-1.00).

Nocturnal and daytime cough were defined by the time periods of 23:00-05:00 and 05:00-23:00, respectively. These periods were determined by observations of both the participants and working

<u>Pilot Active Case finding study</u>

Mask and Sputum Sampling

Each participant wore a modified duckbill face mask (Integrity® 600-300) containing 4 strips of Polyvinyl Alcohol (PVA) produced in house by 3D printing, (Figure S2). This study took place after 3 years' experience with the gelatine filters; these were highly friable and include high background amounts of bacterial DNA (10). These features made them both demanding

to handle and limited the range of usefulness, particularly for detecting other respiratory pathogens (10). In contrast PVA collects impacted bacteria at least as efficiently as gelatine with no background signal and can be dissolved directly in water.  Subjects wore these masks for 30 minutes, were directly observed throughout, and were not required to perform any specific respiratory or vocal manoeuvres but were allowed to cough and phonate at will. If they needed to expectorate they were asked to lift the mask briefly and expectorate into a sputum collection pot.

If they had not produced a sputum sample during mask sampling they were asked to produce a sputum sample once the mask had been removed.

The use of a PVA rather than gelatine in this pilot study reflects the progress our group had made in the intervening time between studies (2015-2018). We realised we could detect significant background bacterial DNA within the gelatine matrix which interfered with several molecular assays (10), particularly those related to other respiratory pathogens, hence the switch to PVA which had no background DNA. Any background DNA in gelatine was taken into account when calculating the limit of detection of the assay (see below).

Participant follow up

Those participants who had Xpert MTB/RIF Ultra positive mask samples were followed up at 6 weeks after the initial screening event. Two had been commenced on TB treatment and one had left the vicinity and was not contactable by telephone. The remaining 5 participants underwent repeated mask and sputum sampling, chest radiograph, bronchoaveloar lavage and CT-PET. All participants were reviewed again at 20 weeks. Repeated Mask, sputum and CT-PET investigations were undertaken.

Mask and Sputum processing

The PVA was dissolved in 5 mls of molecular grade water (Hyclone$^{TM}$, UK (filter sterilised 0.1µm)) in a stomacher bag (Seward, UK) by manual manipulation of the matrix. Once dissolved, it was vortexed using a flat vortex platform for 5minutes to dissolve any microscopic PVA clumps. Two mls of the dissolved material was transfer directly into an Xpert MTB/RIF Ultra cassette, without use of the Xpert sample buffer.

Sputum samples were processed using the Xpert MTB/RIF Ultra manufacturer's instructions for sputum analysis (11).

PET-CT Imaging

Prior to PET-CT imaging, subjects were fasted for 4-6 hours. All patients had a blood glucose of <10 mmol/l (range 5.4-7.1). Following an intravenous injection of 18F FDG (range 0.11-0.15 mCi/kg) and uptake period of 60 minutes, imaging was performed using a Siemens Biograph 40 scanner. Image reconstruction was performed using QCLEAR reconstruction. DICOM images were viewed on a dedicated workstation. Images were interpreted by independent radiology and nuclear medicine consultants in Pretoria and Leicester (UK).

PMA Mask sample analysis at 20 week follow up

Mask samples taken at the 20 week follow up were analysed by Xpert TMB/RIF Ultra following treatment with propidium monoazide (PMA). The dissolved PMA was centrifuged at 13,400 xg for 10 minutes and re-suspended in 500µl molecular grade water (Hyclone$^{TM}$, UK filter (sterilised 0.1µm)) with PMA to a final concentration of 500µM and processed as described by Nikolayevskyy and colleagues (12). Following light treatment, 1.5mls molecular grade water (Hyclone$^{TM}$, UK filter (sterilised 0.1µm)) was added to the sample and loaded directly into the Xpert MTB/RIF Ultra cassette for analysis.

<u>Limit of Detection Methods for IS6110 assay</u>

Mid – exponential Mtb H37Rv underwent serial 10-fold dilution to $10^7$ and filters were contaminated with 100µl of each dilution in 10µl drops across the surface of the filter. Mycobacterial DNA was isolated and extracted before IS6110 copies were quantified. A negative control arm was analysed using un-contaminated filters. This experiment was conducted in technical triplicate. The CFU of original Mtb suspension was calculated using the drop plate method described by Hoben and colleagues (13). The number of Mtb genomes recovered was calculated by dividing the absolute quantification of IS6110 by 16 (IS6110 copy number in H37Rv).

A limit of detection (LoD) for this method was calculated using the following formulae outlined by Armbruster and colleagues (14), the results from the dilution series and a further 9 blank filters which were processed in the same way in order to calculate the limit of the blank (LoB).

$LoB = meanBlank + 1.645(SD_{Blank})$

$LoD = LoB + 1.645(SD_{low\ concentration\ sample})$.

Quantification of Mtb genomes recovered from differing dilutions of contaminated filters using the NaOH and in house method is displayed in Figure S3. The mean (SD) of the IS6110 copies recovered from the 12 bank filters processed was calculated as 218.9(27.2) and so the LoB was calculated as 264. The standard deviation of the lowest dilution that was above the blank (i.e. $1.8x10^3$) was calculated as 162.5.

Therefore LoD was calculated as:

**LoD = 264+1.645(162.5)= 531 or 5.3x1**

Supplemental Table and Figures

| PID | RD9 value | IS6110 Value | Ratio |
|---|---|---|---|
| 1 | 1.3E+03 | 6.7E+03 | 5 |
| 2 | 6.5E+06 | 5.3E+06 | 1 |
| 3 | 2.2E+04 | 6.5E+04 | 3 |
| 4 | 7.3E+07 | 9.7E+08 | 13 |
| 5 | 2.5E+02 | 3.3E+03 | 13 |
| 6 | 1.7E+02 | 1.0E+03 | 6 |
| 7 | 1.1E+08 | 2.1E+08 | 2 |
| 8 | 1.2E+07 | 1.9E+08 | 16 |
| 9 | 9.1E+02 | 1.5E+04 | 16 |
| 10 | 1.2E+03 | 3.4E+04 | 28 |
| 11 | 1.8E+04 | 2.7E+04 | 2 |
| 12 | 1.8E+06 | 6.2E+06 | 3 |
| 13 | 3.3E+08 | 1.2E+09 | 4 |
| 14 | 1.3E+04 | 5.4E+04 | 4 |
| 15 | Failed PCR | 3.1E+04 | Failed PCR |
| 16 | 5.7E+04 | 4.8E+04 | 1 |
| 17 | 1.1E+03 | 2.0E+03 | 2 |
| 18 | 9.2E+02 | 1.3E+03 | 1 |
| 19 | 8.3E+02 | 1.4E+03 | 2 |
| 20 | 8.1E+05 | 1.6E+06 | 2 |
| 21 | 1.9E+07 | 3.6E+08 | 18 |
| 22 | 8.5E+02 | 1.1E+04 | 12 |
| 23 | 4.6E+02 | 1.6E+04 | 36 |
| 24 | 6.3E+03 | 9.7E+03 | 2 |

**Table S1: Comparison of RD9 and IS6110 signals in subset of samples for Participants with calculated ratios**

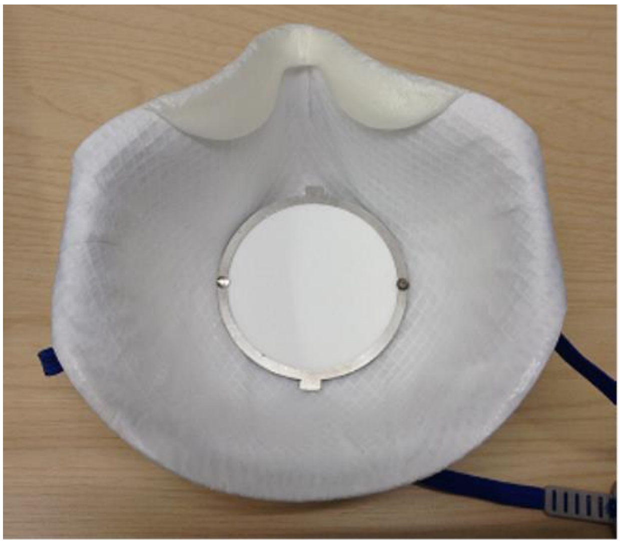

**Figure S1: FPP1 face mask containing a gelatine filter with a diameter of 60mm**

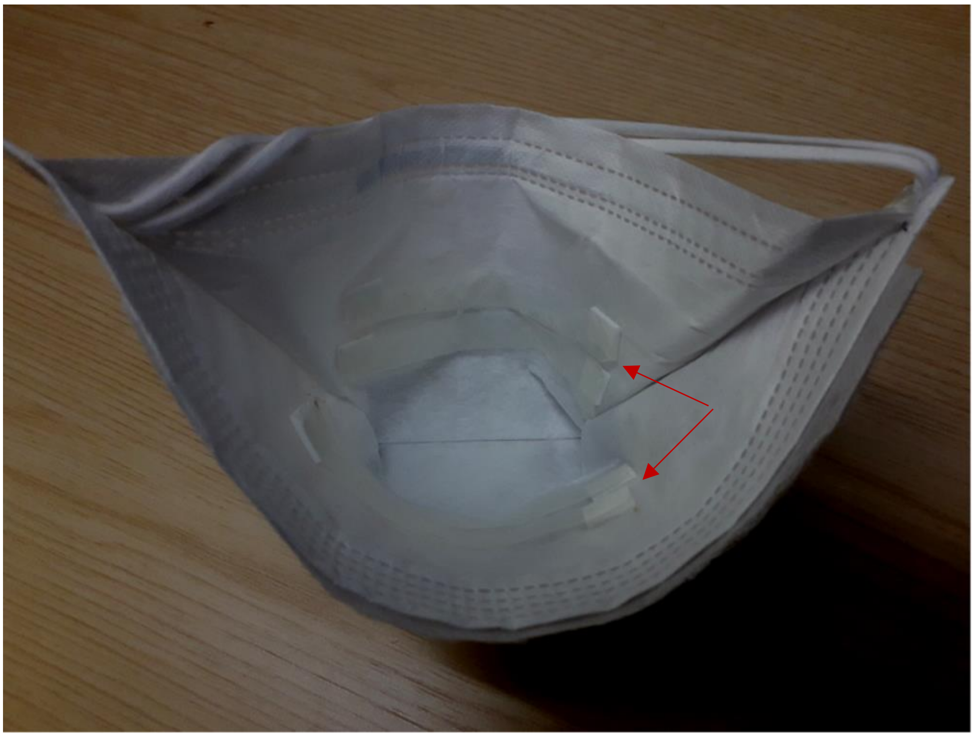

**Figure S2: Duckbilled Face Mask containing 4 strips (arrows) of 3D printed Polyvinyl Alcohol (PVA) each measuring 90mm x 10mm.**

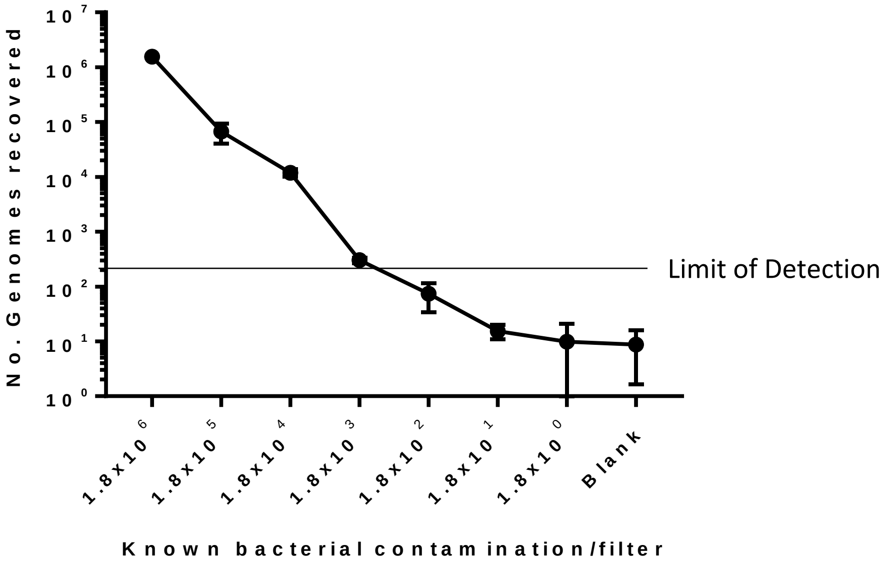


**Figure S3: Dilution series of droplet contamination onto Gelatine filters with Mtb H37Rv in-vitro.** Mean and SD of technical triplicates

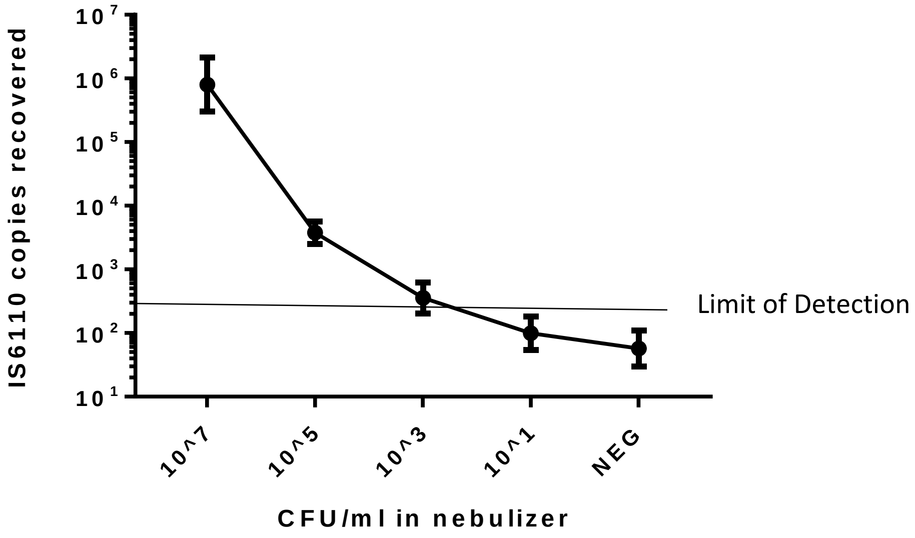


**Figure S4: Dynamic range of filter recovered bacilli.** Recovery of nebulized *M. bovis* BCG Glaxo from gelatine filters exposed for 15 minutes to the bacillary concentrations indicated in a containment system. Mean and SD of biological duplicates and technical triplicates

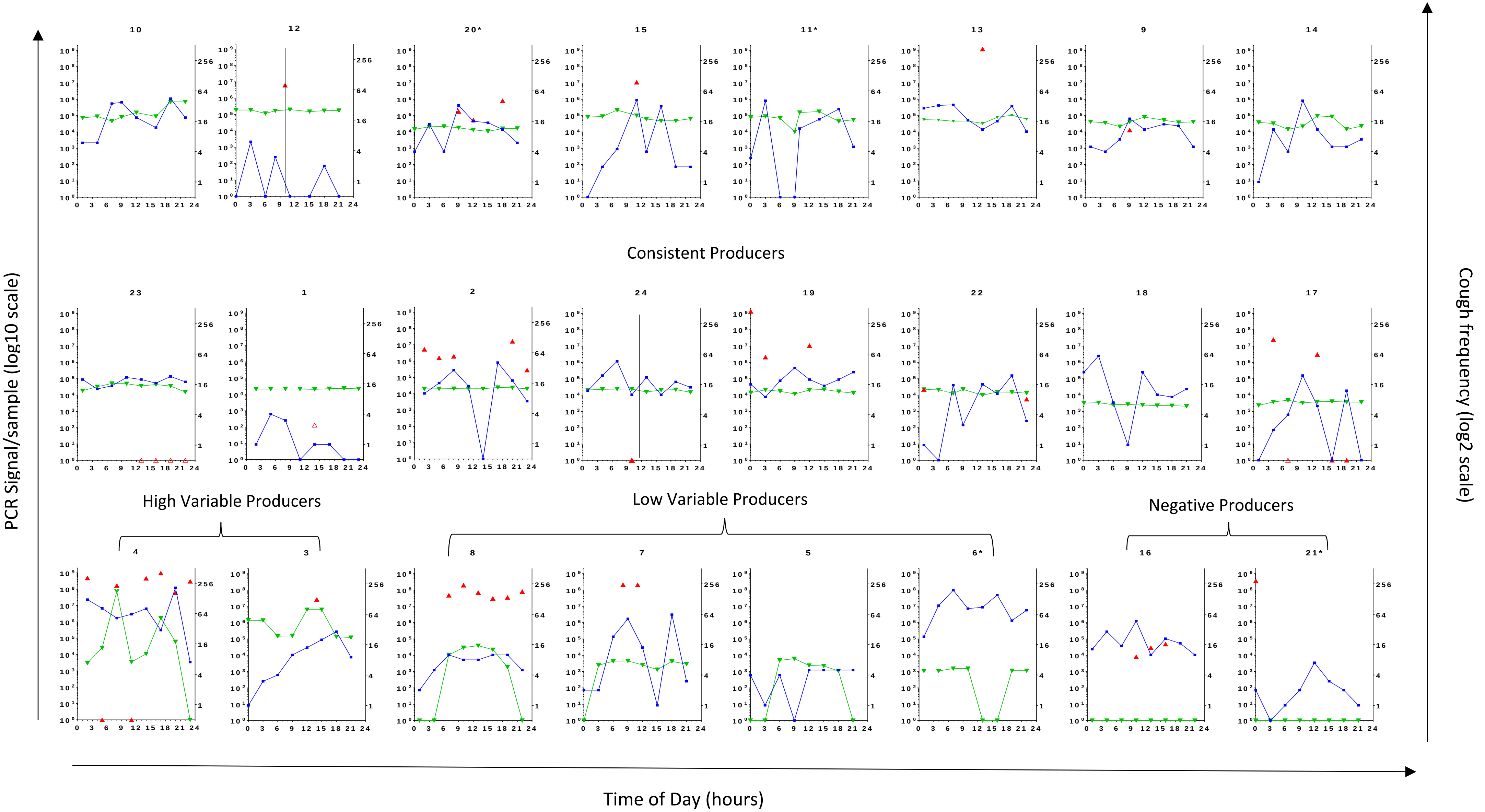


**Figure S5: Pattern of 24 Hour Mycobacterial output by TB patients in Mask and sputum samples compared with cough.**

**Key:** Green –Mask burden – each one hour sample displayed
Red – Sputum burden – collected 3 hourly – missing values = no sputum expectorated, unfilled symbols = samples excluded from analysis due to inadequate processing
Blue – Cough Frequency – coughs/per hour
*HIV negative participants, for 12 &24 the time of treatment initiation during the study is indicated. Plots grouped according to pattern of aerosol production

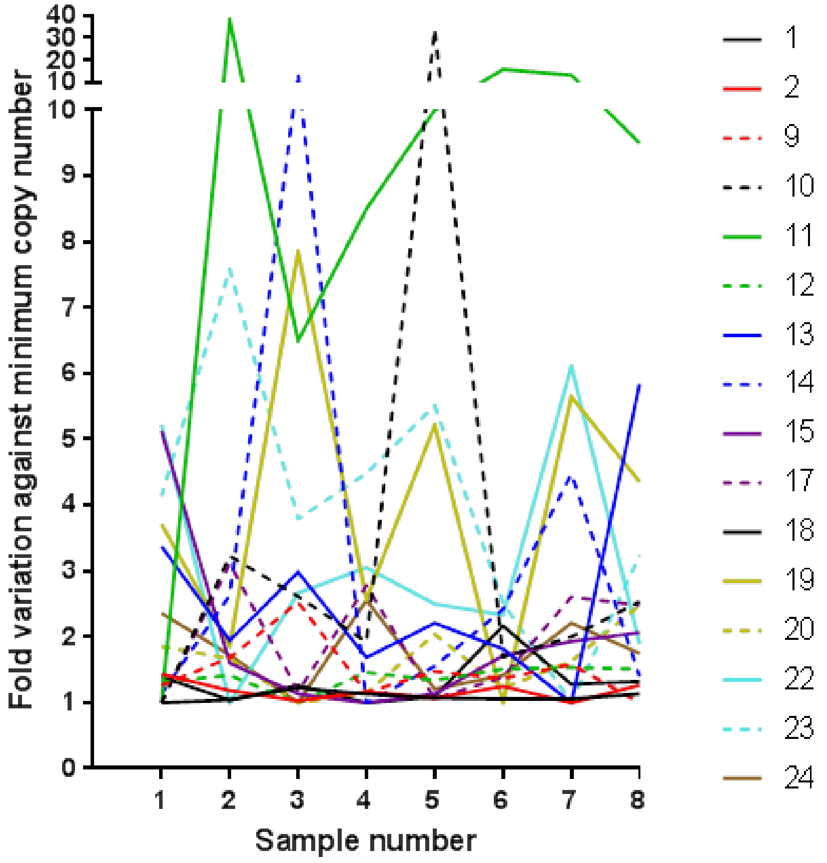


**Figure S6: Variation of PCR signals in the eight mask samples collected for the 16 consistent aerosol producers.**

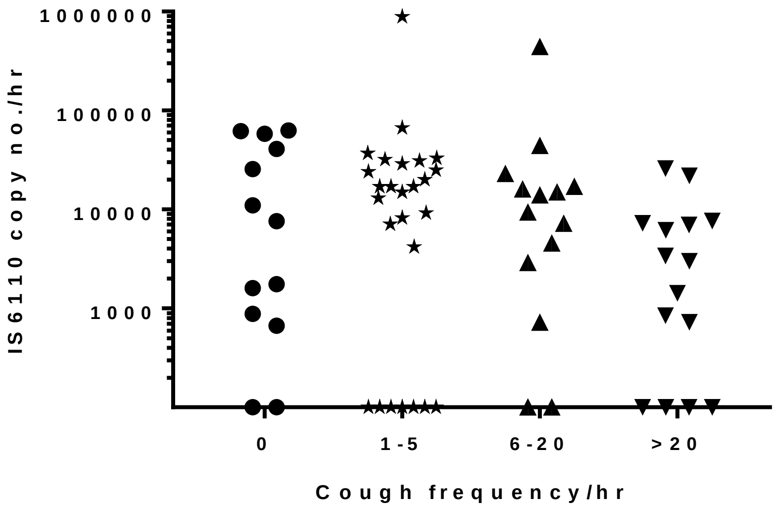


**Figure S7: Mask Mtb burden detected in samples taken when participants were sleeping** grouped by cough recorded during sampling. Note: Mtb levels in samples plotted on *x* axis were below the limit of detection (negative)

| PID | Sample 1 | | Sample 2 | | Sample 3 | | Sample 4 | | Sample 5 | | Sample 6 | | Sample 7 | | Sample 8 | | p-value |
|---|---|---|---|---|---|---|---|---|---|---|---|---|---|---|---|---|---|
| | PCR signal | CF | PCR signal | CF | PCR signal | CF | PCR signal | CF | PCR signal | CF | PCR signal | CF | PCR signal | CF | PCR signal | CF | |
| 1 | 6700 | 16 | 7000 | 2 | 8200 | 1 | 7600 | 0 | 7200 | 17 | 7100 | 1 | 7100 | 6 | 7600 | 0 | 0.13 |
| 2 | 8600 | 28 | 7100 | 3 | 6200 | 38 | 7000 | 41 | 6400 | 19 | 7500 | 4 | 6000 | 4 | 7600 | 14 | 0.9 |
| 3 | 4E+06 | 7 | 46000 | 11 | 45000 | 13 | 37000 | 1 | 9E+05 | 3 | 32000 | 4 | 65000 | 10 | 40000 | 7 | 0.6 |
| 4 | 2E+06 | 111 | 61000 | 293 | 0 | 318 | 3000 | 121 | 26000 | 344 | 7E+07 | 211 | 3500 | 73 | 11000 | 82 | 0.98 |
| 5 | 840 | 5 | 750 | 7 | 730 | 7 | 0 | 7 | 0 | 1 | 0 | 1 | 0 | 4 | 3300 | 2 | 0.57 |
| 6 | 1000 | 147 | 0 | 95 | 0 | 46 | 0 | 81 | 760 | 77 | 0 | 37 | 730 | 39 | 0 | 193 | 0.88 |
| 7 | 0 | 42 | 890 | 0 | 1900 | 47 | 0 | 2 | 0 | 2 | 1600 | 0 | 1300 | 23 | 1700 | 27 | 0.47 |
| 8 | 7200 | 6 | 12000 | 11 | 13000 | 9 | 1300 | 10 | 0 | 12 | 0 | 2 | 0 | 5 | 0 | 9 | 0.56 |
| 9 | 12000 | 15 | 16000 | 8 | 24000 | 14 | 11000 | 23 | 14000 | 12 | 13000 | 1 | 15000 | 4 | 9500 | 3 | 0.99 |
| 10 | 13000 | 65 | 42000 | 13 | 34000 | 12 | 25000 | 2 | 4E+05 | 19 | 22000 | 23 | 26000 | 6 | 33000 | 45 | 0.58 |
| 11 | 2000 | 1 | 76000 | 8 | 13000 | 34 | 17000 | 5 | 20000 | 3 | 32000 | 0 | 27000 | 114 | 19000 | 0 | 0.90 |
| 12 | 54000 | 0 | 58000 | 0 | 41000 | 0 | 60000 | 2 | 55000 | 0 | 62000 | 0 | 63000 | 0 | 62000 | 0 | 0.99 |
| 13 | 26000 | 17 | 15000 | 11 | 23000 | 18 | 13000 | 6 | 17000 | 10 | 14000 | 12 | 7700 | 21 | 45000 | 39 | 0.36 |
| 14 | 4300 | 41 | 10000 | 11 | 10000 | 11 | 3800 | 9 | 5900 | 7 | 9200 | 1 | 17000 | 11 | 5300 | 4 | 0.43 |
| 15 | 77000 | 12 | 24000 | 4 | 17000 | 3 | 15000 | 14 | 17000 | 1 | 25700 | 0 | 29000 | 2 | 31000 | 3 | 0.86 |
| 16 | 0 | 103 | 0 | 54 | 0 | 35 | 0 | 3 | 0 | 1 | 0 | 17 | 0 | 32 | 0 | 33 | 1.00 |
| 17 | 633 | 10 | 1970 | 13 | 740 | 17 | 1760 | 0 | 667 | 0 | 880 | 0 | 1650 | 4 | 1570 | 1 | 0.99 |
| 18 | 920 | 25 | 687 | 9 | 833 | 12 | 660 | 0 | 720 | 13 | 1430 | 28 | 847 | 59 | 873 | 7 | 0.12 |
| 19 | 5200 | 34 | 2600 | 20 | 11000 | 14 | 3600 | 11 | 7300 | 6 | 1400 | 16 | 7900 | 8 | 6100 | 48 | 0.36 |
| 20 | 50000 | 33 | 45000 | 34 | 27000 | 22 | 31000 | 15 | 55000 | 4 | 33000 | 1 | 44000 | 14 | 67000 | 3 | 0.66 |
| 21 | 0 | 3 | 0 | 9 | 0 | 0 | 0 | 2 | 0 | 8 | 0 | 4 | 0 | 0 | 0 | 0 | 1.00 |
| 22 | 9400 | 2 | 1800 | 1 | 4800 | 5 | 5500 | 14 | 4500 | 0 | 4200 | 5 | 11000 | 0 | 3400 | 31 | 0.52 |
| 23 | 12000 | 22 | 22000 | 17 | 11000 | 4 | 13000 | 13 | 16000 | 15 | 7300 | 48 | 2900 | 13 | 9400 | 15 | 0.85 |
| 24 | 8920 | 0 | 6520 | 2 | 3780 | 10 | 9670 | 32 | 4570 | 14 | 5350 | 18 | 8350 | 62 | 6610 | 46 | 0.58 |

**Table S2: Within-participant associations between cough and mask Mtb burden**

| Criteria | Correlation coefficient (95% CI) | p-value |
|---|---|---|
| **Univariate Analysis** | | |
| Age | 0.32 (-0.11 - 0.65) | 0.13 |
| Gender | | **0.04** |
| HIV status | | 0.63 |
| CD4 count * | 0.06 (-0.41 - 0.50) | 0.81 |
| Duration of Symptoms (weeks) | 0.21 (-0.23 - 0.57) | 0.34 |
| CXR Grade# | 0.46 (0.04 - 0.74) | **0.03** |
| Presence of cavitations on CXR# | | 0.88 |
| Sputum AFB Grade¥ | 0.56 (0.11 - 0.82) | **0.01** |
| Sputum Xpert Grade | 0.26 (-0.17 - 0.61) | 0.22 |
| Sputum Culture TTP$ | -0.50 (-0.80 - -0.01) | **0.04** |
| Patient perception of cough severity (VAS)β | 0.50 (0.09 - 0.80) | **0.02** |
| 24 hour quantity PCR Signals in mask sampling | 0.10 (-0.33 - 0.50) | 0.63 |
| 24 hour quantity PCR Signals in Sputum§ | -0.002 (-0.44- 0.44) | 0.99 |
| **Multivariate Analysis** | | |
| Model | $R^2$= 41% | 0.06 |
| Gender | 0.2 (-505.0 - 545.50) | 0.93 |
| Sputum culture TTP$ | -0.23 (-108.62 - 48.86) | 0.42 |
| Sputum AFB Grade¥ | 0.52 (-25.40 - 387.57) | 0.08 |
| Patient perception of cough (VAS)β | 0.36 (-26.47 - 154.40) | 0.15 |
| CXR Grade# | 0.42 (-11.25 - 9.73) | 0.86 |

**Table S3: Criteria associated Cough Frequency**. Data represents the ability of all variables in this study to predict cough frequency using Spearman's correlation co-efficient for continuous data and Mann Whitney U for categorical. Multivariate analysis was carried out using variables that were statistically significant; Gender, Time To Positivity in liquid sputum culture, Sputum AFB grade, Patient perception of cough and Chest x-ray grade. §Total sputum output over 24 hours analysed in 21 pts. * CD4 recorded for all 20 HIV positive pts # CXR grade and presence of cavities for 23 pts ¥ Sputum AFB grade available for 17 pts $Sputum culture TTP results available for 16 pts β VAS recorded for 22 pts.

1. Turapov O, O'Connor BD, Sarybaeva AA, Williams C, Patel H, Kadyrov AS, Sarybaev AS, Woltmann G, Barer MR, Mukamolova GV. Phenotypically Adapted Mycobacterium tuberculosis Populations from Sputum Are Tolerant to First-Line Drugs. *Antimicrob Agents Chemother* 2016; 60: 2476-2483.
2. C.A. Reddy TJB, J.A. Breznak, G.A. Marzluf, T.M.Schmidt, L.R. Snyder. Methods for General and Molecular Microbiology. ASM Press; 2007.
3. Akkerman OW, van der Werf TS, de Boer M, de Beer JL, Rahim Z, Rossen JW, van Soolingen D, Kerstjens HA, van der Zanden AG. Comparison of 14 molecular assays for detection of Mycobacterium tuberculosis complex in bronchoalveolar lavage fluid. *J Clin Microbiol* 2013; 51: 3505-3511.
4. Dorak MT. Real Time PCR Taylor & Francis Group; 2006.
5. Chae H, Han SJ, Kim SY, Ki C-S, Huh HJ, Yong D, Koh W-J, Shin SJJJocm. Development of a one-step multiplex PCR assay for differential detection of major Mycobacterium species. 2017: JCM. 00549-00517.
6. Birring SS, Fleming T, Matos S, Raj AA, Evans DH, Pavord ID. The Leicester Cough Monitor: preliminary validation of an automated cough detection system in chronic cough. *Eur Respir J* 2008; 31: 1013-1018.
7. Sinha A, Lee KK, Rafferty GF, Yousaf N, Pavord ID, Galloway J, Birring SS. Predictors of objective cough frequency in pulmonary sarcoidosis. *Eur Respir J* 2016; 47: 1461-1471.
8. Turner RD, Birring SS, Darmalingam M, Hooper RL, Kunst H, Matos S, Bothamley GH. Daily cough frequency in tuberculosis and association with household infection. *Int J Tuberc Lung Dis* 2018; 22: 863-870.
9. Chan KK, Ing AJ, Laks L, Cossa G, Rogers P, Birring SS. Chronic cough in patients with sleep-disordered breathing. *Eur Respir J* 2010; 35: 368-372.
10. Kennedy M, Ramsheh MY, Williams CML, Auty J, Haldar K, Abdulwhhab M, Brightling CE, Barer MR. Face mask sampling reveals antimicrobial resistance genes in exhaled aerosols from patients with chronic obstructive pulmonary disease and healthy volunteers. *BMJ open respiratory research* 2018; 5: e000321.
11. Chakravorty S, Simmons AM, Rowneki M, Parmar H, Cao Y, Ryan J, Banada PP, Deshpande S, Shenai S, Gall A, Glass J, Krieswirth B, Schumacher SG, Nabeta P, Tukvadze N, Rodrigues C, Skrahina A, Tagliani E, Cirillo DM, Davidow A, Denkinger CM, Persing D, Kwiatkowski R, Jones M, Alland D. The New Xpert MTB/RIF Ultra: Improving Detection of Mycobacterium tuberculosis and Resistance to Rifampin in an Assay Suitable for Point-of-Care Testing. *mBio* 2017; 8.
12. Nikolayevskyy V, Miotto P, Pimkina E, Balabanova Y, Kontsevaya I, Ignatyeva O, Ambrosi A, Skenders G, Ambrozaitis A, Kovalyov A, Sadykhova A, Simak T, Kritsky A, Mironova S, Tikhonova O, Dubrovskaya Y, Rodionova Y, Cirillo D, Drobniewski F. Utility of propidium monoazide viability assay as a biomarker for a tuberculosis disease. *Tuberculosis (Edinb)* 2015; 95: 179-185.
13. Hoben H, Somasegaran PJA, microbiology e. Comparison of the pour, spread, and drop plate methods for enumeration of Rhizobium spp. in inoculants made from presterilized peat. 1982; 44: 1246-1247.
14. Armbruster DA, Pry T. Limit of blank, limit of detection and limit of quantitation. *Clin Biochem Rev* 2008; 29 Suppl 1: S49-52.
